# Metabolic phenotype of methylmalonic acidemia in mice and humans: the role of skeletal muscle

**DOI:** 10.1186/1471-2350-8-64

**Published:** 2007-10-15

**Authors:** Randy J Chandler, Jennifer Sloan, Hong Fu, Matthew Tsai, Sally Stabler, Robert Allen, Klaus H Kaestner, Haig H Kazazian, Charles P Venditti

**Affiliations:** 1 Genetic Diseases Research Branch, National Human Genome Research Institute, National Institutes of Health, Bethesda MD 20892 USA; 2 Department of Genetics, University of Pennsylvania School of Medicine, Philadelphia, PA 19114, USA; 3 Department of Medicine, University of Colorado School of Medicine, Denver CO 80206, USA

## Abstract

**Background:**

Mutations in methylmalonyl-CoA mutase cause methylmalonic acidemia, a common organic aciduria. Current treatment regimens rely on dietary management and, in severely affected patients, liver or combined liver-kidney transplantation. For undetermined reasons, transplantation does not correct the biochemical phenotype.

**Methods:**

To study the metabolic disturbances seen in this disorder, we have created a murine model with a null allele at the methylmalonyl-CoA mutase locus and correlated the results observed in the knock-out mice to patient data. To gain insight into the origin and magnitude of methylmalonic acid (MMA) production in humans with methylmalonyl-CoA mutase deficiency, we evaluated two methylmalonic acidemia patients who had received different variants of combined liver-kidney transplants, one with a complete liver replacement-kidney transplant and the other with an auxiliary liver graft-kidney transplant, and compared their metabolite production to four untransplanted patients with intact renal function.

**Results:**

Enzymatic, Western and Northern analyses demonstrated that the targeted allele was null and correctable by lentiviral complementation. Metabolite studies defined the magnitude and tempo of plasma MMA concentrations in the mice. Before a fatal metabolic crisis developed in the first 24–48 hours, the methylmalonic acid content per gram wet-weight was massively elevated in the skeletal muscle as well as the kidneys, liver and brain. Near the end of life, extreme elevations in tissue MMA were present primarily in the liver. The transplant patients studied when well and on dietary therapy, displayed massive elevations of MMA in the plasma and urine, comparable to the levels seen in the untransplanted patients with similar enzymatic phenotypes and dietary regimens.

**Conclusion:**

The combined observations from the murine metabolite studies and patient investigations indicate that during homeostasis, a large portion of circulating MMA has an extra-heptorenal origin and likely derives from the skeletal muscle. Our studies suggest that modulating skeletal muscle metabolism may represent a strategy to increase metabolic capacity in methylmalonic acidemia as well as other organic acidurias. This mouse model will be useful for further investigations exploring disease mechanisms and therapeutic interventions in methylmalonic acidemia, a devastating disorder of intermediary metabolism.

## Background

Methylmalonic acidemia is the biochemical hallmark of a group of autosomal recessive genetic metabolic disorders that prevent the body from converting L-methylmalonyl-CoA into succinyl-CoA[1]. These disorders are caused by mutations in the methylmalonyl-CoA mutase apoenzyme or by impaired synthesis of the enzymatic cofactor, 5'deoxyadenosylcobalamin [2,3]. Affected individuals display massive elevations of methylmalonic acid (MMA) in body tissues and fluids, have a well-recognized metabolic phenotype characterized by extreme metabolic fragility, and a poor outcome marked by early mortality and substantial lifelong morbidity [4-8].

Mutations in the methylmalonyl-CoA mutase (*MUT*) gene are the cause of *mut *class methylmalonic acidemia. Two enzymatic phenotypes within this class are recognized. In *mut*^*o*^, there is no detectable or residual enzyme activity. In *mut*^-^, enzyme activity is markedly reduced but can sometimes be stimulated *in vitro *with supraphysiological concentrations of vitamin B12 [4,9-11]. The correlation between *in vitro *and *in vivo *vitamin B12 responsiveness in patients classified as *mut*^- ^is uncertain, since most do not demonstrate a physiological response to parenteral cobalamin [12-14]. Typically, patients with *mut*^*o *^methylmalonic acidemia display more severe biochemical and clinical phenotypes than patients who harbor cofactor responsive lesions [4]. In a recent clinical study, vitamin B12 unresponsive patients had a median survival of six years [7].

The treatment of isolated methylmalonic acidemia was established in the 1960s and consists of alkali replacement and nutritional management, mainly designed to limit amino acid precursors [15-17]. Carnitine supplementation and intermittent antibiotic therapy are newer measures, whose efficacy has not been determined [18-21]. The precise etiology of the complications of methylmalonic acidemia is uncertain, and even well-treated patients remain at risk for intermittent decompensation, pancreatitis, metabolic infarction of the basal ganglia, and renal failure[6-8,22-24].

Solid organ transplantation has been used as a therapy for methylmalonic acidemia and a few patients have received liver, kidney, and combined liver-kidney transplants [25-34]. Liver and liver-kidney transplant recipients do not experience ketoacidotic attacks, but remain at risk for renal disease[29] and metabolic infarction of the basal ganglia [30]. They also exhibit persistent methylmalonic acidemia/aciduria [33] and may have extreme elevations of MMA in the cerebrospinal fluid (CSF) [34]. The origin of MMA production and the magnitude of MMA elevations in transplant patients have not been carefully studied.

We created a mouse model of *mut*^*o *^methylmalonic acidemia to investigate the pathophysiological mechanisms of the disease and study the etiology of MMA production in the disorder. In this report, we describe the metabolic phenotype of the *mut*^*o *^animals and compare it to the human condition in the post-transplant state. Our data suggest that the extra-hepatorenal metabolism of propionyl-CoA provides the major pool of metabolites seen in methylmalonic acidemia and highlights the role that skeletal muscle plays in the pathophysiology of this common organic aciduria.

## Methods

### Construction of the exon 2 floxed, exon 3 deleted methylmalonyl-CoA mutase allele

A 129 Sv/Ev BAC clone containing the murine methylmalonyl-CoA mutase gene was used to prepare a targeting construct. In brief, a set of contiguous EcoRI restriction fragments containing the 5'end of the gene and first through fourth exons was assembled in pBluescript. The first contained a fragment that harbored the second and third exons of the gene. A loxP linker was introduced into the 5' EcoRI site. The linker carries a unique ApaLI site. The two 5' genomic EcoRI fragments were added sequentially, followed by the addition of a PstI fragment containing a portion of exon 3 to the 3' end. A cassette derived from pHR1 that carried a loxP site, the pMC1 promoter, the neomycin resistance gene, and the Sv40 poly-A signal was placed in between the unique AvrII site and the Bsu36I site in the targeting construct[35]. A diphtheria toxin-A fragment cassette under the control of the pMC1 promoter was next inserted at the 5' end of the construct in the reverse orientation to generate the targeting construct[36]. ES clones were screened using combination of long-range PCR/enzyme digestion and Southern blotting. Primers that flank the 5' loxP site were used to genotype the animals. (See additional file 1 and additional file 2 for the representative results, PCR conditions, and primer sequences). The National Human Genome Research Institute Animal Care and Use Committee approved the animal experiments.

### Biochemical Characterization and Lentiviral Complementation

*Mut *mutant animals were identified by genotyping and metabolic analysis using the para-nitroanaline reaction (See additional file 1 for representative results of metabolic screening) [37]. Organs were harvested and nucleic acids extracted using the DNeasy Tissue kit and RNeasy Total RNA Isolation from Lipid Tissues kit (Qiagen). The Northern blot was performed with the Northern Max-Gly kit (Ambion) following the manufacturer's protocol. 20 μg of total RNA, derived from the liver of *Mut *null or wild-type neonatal mice, was used for Northern blotting. The probe for the mouse gene was a portion of the cDNA, located between the start codon at the 5' end and an NcoI site at position 1187, encoding exons 1–5. The blot was stripped and probed with an actin cDNA, which was provided by Ambion as a loading control.

Whole cell extracts from the liver and murine embryonic fibroblast cell lines were analyzed by immunoblotting and were probed with rabbit polyclonal antisera raised against the murine methylmalonyl-CoA mutase enzyme [38] or beta-actin (Abcam, Cambridge, MA). The anti-mutase antibody was used at a dilution of 1:750, and beta-actin was used at 1:5,000. Goat anti-rabbit (Chemicon, Temecula, CA) was used as a secondary antibody at a dilution of 1:10,000. Chemiluminescent detection was performed with reagents from Pierce. Recombinant murine methylmalonyl-CoA mutase enzyme [39] was used to verify the specificity of the rabbit anti-mutase antisera and to provide a positive control in Western blot analysis experiments.

Methylmalonyl-CoA mutase activity was determined indirectly by measuring [1-^14^C] propionate incorporation into macromolecules as described[40]. All propionate incorporation assays were performed in triplicate. [1-^14^C] Sodium propionate was purchased (Perkin Elmer, Cambridge, MA) as a custom preparation at a specific activity of 55.0 mCi/mmol (2 mCi/ml).

Murine embryonic fibroblast cell lines were prepared after harvesting embryos on day E12.5. The cell lines were genotyped and expanded prior to use. A recombinant lentivirus that expressed the murine methylmalonyl-CoA mutase was prepared by cloning a sequence-verified, full-length murine methylmalonyl-CoA mutase cDNA into pLenti6 (Invitrogen, Carlsbad, CA). The lentiviral construct has the *Mut *gene driven by the CMV promoter and the backbone carries a blasticidin cassette driven by the E7 promoter. A control lentivirus expressing EGFP cloned in an identical fashion was also prepared and used in parallel for correction experiments.

All viral stocks were prepared according to manufacturers instructions. *Mut *null MEF cell lines were infected with lentiviral stocks expressing either murine methylmalonyl-CoA mutase or eGFP. The transduced MEFs were selected and expanded on DMEM with 5 percent fetal bovine serum containing 2.5 μg/ml blasticidin prior to [1-^14^C] propionate incorporation studies.

### Metabolic Studies

Plasma was isolated from animals immediately after euthanization by collecting blood in tubes that contained 1–5 μl of diluted sodium heparin. The samples were immediately centrifuged, the plasma removed, diluted in water, and stored at -80°C in a screw top tube for later analysis. The mice underwent gentle bladder massage to produce 1–10 μl of urine that was immediately analyzed or diluted and stored in a similar fashion as the plasma. Organ dissections were performed under a dissecting microscope to remove the brain, liver, skeletal muscle, and kidneys. Organs were weighed and snap frozen prior to storage at -80°C and subsequent processing.

Two methods were used to measure MMA in the mice. Plasma, urine, and organ extracts were analyzed by gas chromatography-mass spectrometry with stable isotopic internal calibration to measure MMA and 2-methylcitrate isomers I and II[41]. The para-nitroanaline reaction was used for rapid identification of affected animals[37].

### Patient Studies

Patient studies were conducted in compliance with the Helsinki Declaration and were approved by the National Human Genome Research Institute Institutional Review Board as part of NIH study 04-HG-0127 "Clinical and Basic Investigations of Methylmalonic Acidemia and Related Disorders" after informed consent was obtained. Metabolic parameters and creatinine clearance were measured during two consecutive 24-hour urine collections and were used to estimate the glomerular filtration rate (GFR) and MMA output.

Two patients had undergone combined liver-kidney transplantation as a treatment for *mut*^*o *^class methylmalonic acidemia prior to enrollment. Briefly, the patient labeled L(A)KT, a 19-year old Caucasian female, was diagnosed on the second day of life following an episode of hypothermia and lethargy. At age 12, she received a cadaveric renal transplant and subhepatic heterotopic auxiliary liver transplant[28]. At the time of these studies, she was receiving 0.8 g/kg/d whole protein. Patient LKT, a 27-year old Mexican-American female, was diagnosed in the neonatal period. At age 22, she received an orthotopic liver transplant followed by a kidney transplant two years later[29]. At the time of these studies, she was receiving 0.5 g/kg/d whole protein. The other patients (N = 4) ranged in age from 4 to 21 years and have not received transplants. All patients harbored complementation status-defined *mut*^*o *^class lesions and were selected as a comparator group because they had identical enzymatic phenotypes and importantly, preserved renal function, with measured creatinine clearances greater than 40 percent of predicted. All patients were well at the time of hospitalization and were individually prescribed high energy, low protein diets that provided between 0.3–0.9 g/kg/d whole protein. One patient (Pt 4) also received supplemental isoleucine.

### Statistics

Statistical manipulations were carried out using Statistical Analysis Software (SAS) version 9 (SAS Institute, Cary, North Carolina, USA). Differences in the plasma and urinary MMA concentrations between mutant mice over time and patient groups was determined by one-way ANOVA and adjusted for multiple comparisons using the Tukey-Kramer method. Tissue metabolites were analyzed using a mixed model ANOVA to account for both random and fixed effects. The model included the effects of time, organ, and organ by time interaction. p-values for the higher-level interaction comparisons were adjusted using bootstrap simulation[42]. p-values of 0.05 or less were considered statistically significant.

## Results

### Methylmalonyl-CoA Mutase Mutant Mice

**Figure 1:**
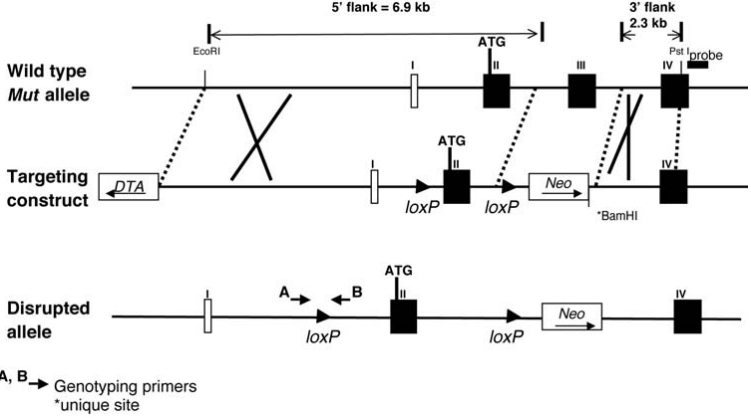
Targeting construct and null allele. (A) An overview of the targeting construct and disrupted allele. Selected restriction sites are indicated. The initiator codon lies in the second coding exon and the coenzyme A binding pocket in the third coding exon. Recombination sites (loxP), the diptheria toxin A cassette (DTA) and neomycin resistance gene (NEO) are shown. The location of genotyping primers that flank the 5' loxP site are indicated by A and B.

**Figure 2:**
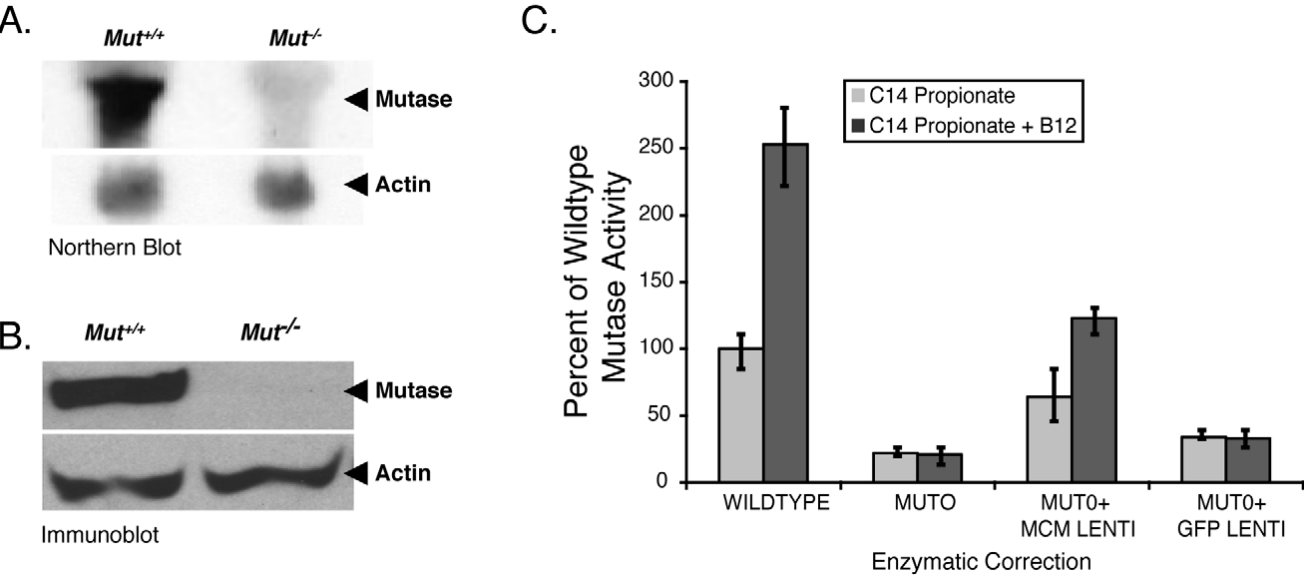
Northern, Western, enzymatic and correction studies. (A) Northern analysis of total RNA extracted from wild-type and *Mut *knockout livers. The mutase message is apparent in the wild-type sample and absent in the *Mut *mutant liver. Actin hybridization after stripping and reprobing shows the RNA to be intact and equally reactive. (B) Western blotting using anti-mutase antisera reveals a band of ~80 kd in the wild-type liver extracts that is completely absent from the mutant liver extracts. (C) [1-^14^C] propionic acid incorporation, with and without vitamin B12, in various cell lines, expressed as percent of wild-type activity. The wild type activity varied between days and ranged from 5.0–15.0 nmol C14 propionate/mg protein/18 hrs. Error bars surround the standard deviation from triplicate measurements. The corrected *Mut *null murine cell line (labeled MUTO + MCM LENTI) shows restored propionate flux when compared with the *Mut *null and GFP-transduced cell lines.

Homologous recombination in mouse embryonic stem cells was used to engineer a deletion in the third exon of the methylmalonyl-CoA mutase (*Mut*) gene and to flank the exon 2 with loxP sites to create a versatile null allele, suitable for Cre-mediated genomic manipulations [43] (Figure 1). RNA and enzymatic assays verified that the *Mut *mutant allele abolished the function of the methylmalonyl-CoA mutase enzyme (Figure 2). Total RNA was isolated from the livers of *Mut *deficient mice and wild-type controls. Northern blot experiments using a portion of the murine cDNA encompassing the first through fifth exons as a probe showed no hybridization signal in *Mut *null liver RNA (Figure 2A). The wild type sample had a large and strongly reactive band of approximately 3.0 kb. RT-PCR experiments yielded no product in *Mut *null liver RNA when amplified with primers that flanked the deletion (Exons 2–6) or were anchored within the deletion (Exons 3–4) (data not shown, see additional file 2 for primer sequences and RT-PCR conditions). Actin hybridization (Figure 2A) and amplification of GAPDH by RT-PCR on both samples confirmed that the RNA was intact (data not shown). Western blotting using an anti-mutase antibody revealed complete loss of protein in the liver extracts (Figure 2B).

Murine embryonic fibroblasts (MEF) derived from wild-type and *Mut *mutant embryos were isolated and used to construct cell lines. The cells were genotyped and used to assess the functional activity of methylmalonyl-CoA mutase by [1-^14^C]propionate incorporation and responsiveness to vitamin B12. Compared to wild-type cells, the *Mut *knock-out cell lines were devoid of methylmalonyl-CoA mutase protein (see additional file 3), incorporated very low levels of [1-^14^C] propionate into macromolecules, and exhibited no stimulation by exogenous vitamin B12 (Figure 2C).

The cell lines were then studied in complementation experiments. The full length *Mut *cDNA was placed behind the CMV promoter in a lentivirus that carried an independently expressed blasticidin-resistance cassette and was used to produce viral stocks. Integrated proviruses expressed both genes, and blasticidin selection was used to enrich for transduced cells. [1-^14^C] propionate incorporation showed an increase in mutase activity to near wild-type levels in the transduced *Mut *null MEF cell lines (Figure 2C) whereas a control GFP did not.

### Metabolic Studies in the Mut mutant Mice

In the neonatal period, the *Mut *null animals appeared well until 12 to 24 hours of life, when the size of the milk spot in the stomach decreased and dehydration occurred. The mice typically perished 24 to 36 hours after birth with respiratory distress and non-necrotic lipidotic changes in the liver. Two of the eight *Mut *null mice that were examined had pulmonary hemorrhages, but most had nonspecific post-mortem findings. Increased intracranial pressure was suggested in some animals because the brain appeared compressed against the dura mater on histological examination. Organic acids and amino acids from the plasma and urine were analyzed throughout the life of the mutant and control littermates.

**Figure 3:**
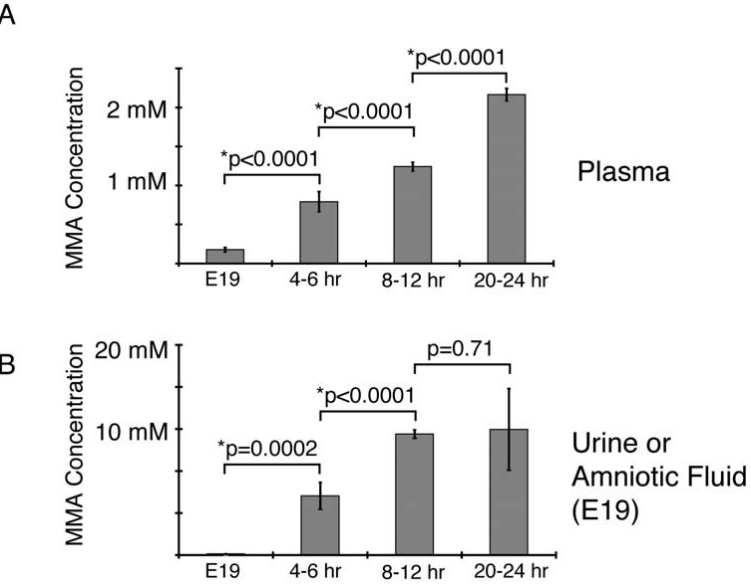
Pre- and postnatal plasma and urine methylmalonic acid levels. (A) MMA concentrations (μM) measured in the plasma over time. Error bars surround the standard deviation. Wild-type (N = 4–8 age matched littermates at each time point, not displayed), *Mut *null prenatal [E19] (N = 3), *Mut *null postnatal 4–6 hours (N = 4), *Mut *null postnatal 8–12 hours (N = 4), and *Mut *null postnatal 20–24 hours (N = 4). Low-level wild-type values not displayed. An asterisk * designates a significant p-value, calculated using a one-way ANOVA with Tukey-Kramer adjustment, for comparison between advancing time points. Prenatal (value = 176 μM) vs. 4–6 hours [F (1,6) = 107; p < 0.0001], 4–6 hr vs 8–12 hours [F (1,6) = 54; p < 0.0001] and 8–12 hours vs 20–24 hours [F (1,4) = 152; p < 0.0001]. At all time points, the mutants were different from the unaffected littermates with p-values less than 0.001. (B) MMA concentrations (mM) in urine or amniotic fluid on day E19 (value = 117 μM) from the samples above. An asterisk * designates a significant p-value, calculated using a one-way ANOVA with Tukey-Kramer adjustment, for comparison between advancing time points. Prenatal vs. 4–6 hours [F (1,6) = 30; p = 0.0002] and 4–6 hr vs 8–12 hours [F (1,6) = 49; p < 0.0001]. However, at 8–12 hours vs 20–24 hours the differences were not significant [F (1,4) = 0.14; p = 0.7143]. In all cases, the mutants were different from the unaffected littermates (not displayed) with p-values less than 0.001.

On several occasions, embryos were harvested on day 16 of gestation to examine the distribution of genotypes and look for possible prenatal manifestations of methylmalonic acidemia, such as growth retardation and malformation. In the four litters examined, mice were present in approximately 1:2:1 ratios, and homozygous *Mut *mutant animals were indistinguishable from heterozygous and homozygous wild-type (WT) animals. Plasma methylmalonic acid concentrations in the heterozygous and homozygous WT animals, measured on embryonic day 19, were equal at the lower limit of assay detection in diluted samples. In contrast, the *Mut *mutant animals demonstrated significant elevations of plasma MMA, with an average concentration of 176 μM (Figure 3A). Amniotic fluid also showed increased methylmalonic acid in the *Mut *mutant (average = 117 μM) compared to wild-type animals (average = 5.3 μM) (Figure 3B).

*Mut *mutant and control animals were harvested at approximately 4 to 6 hours of life for metabolite studies. All the animals had similar weights, developed milk spots, and appeared vigorous. Heterozygous and WT animals had identical biochemical parameters with diluted plasma MMA concentrations near the lower limits of assay detection, but the *Mut *null mice displayed plasma MMA levels in the 700 μM range (Figure 3A) with massive methylmalonic aciduria (Figure 3B).

At 8 to 12 hours of life, while still well and feeding, the neonatal *Mut *animals displayed increasing methylmalonic acidemia and methylmalonic aciduria (Figure 3), with plasma MMA concentrations in the 1.2 mM range and urine MMA levels greater than 20 mM. Control littermates had plasma MMA levels similar to those measured at earlier times.

At the end of life, the *Mut *null mice had plasma MMA concentrations of 2.2 mM, which were not drastically higher than those of the neonatal animals at 8 to 12 hours of life (Figure 3A). As the mice developed agonal respirations, the plasma MMA concentrations rose to greater than 2 mM. Although the animals appeared in extremis, a frank acid/base imbalance caused by MMA seems unlikely, given the plasma MMA concentration at this time.

The pattern of increases in the concentration in the plasma was highly significant within the mutant groups over time: prenatal vs. 4–6 hours [F (1,6) = 107; p < 0.0001], 4–6 hr vs 8–12 hours [F (1,6) = 54; p < 0.0001] and 8–12 hours vs 20–24 hours [F (1,4) = 152; p < 0.0001]. The urinary metabolites showed a similar pattern with prenatal vs. 4–6 hours [F (1,6) = 30; p = 0.0002] and 4–6 hr vs 8–12 hours [F (1,6) = 49; p < 0.0001]. However, at 8–12 hours vs 20–24 hours the differences were not significant [F (1,4) = 0.14; p = 0.7143].

**Figure 4:**
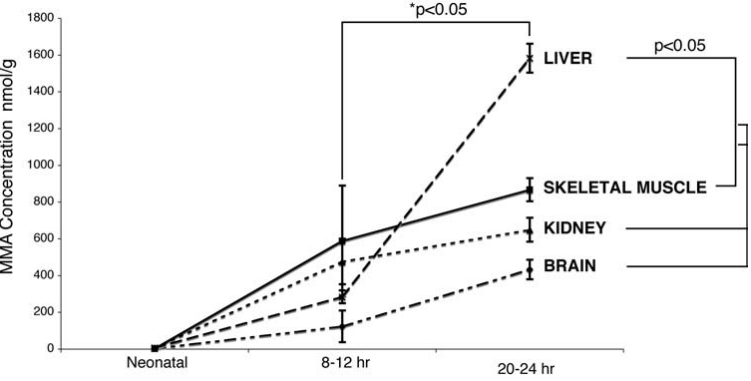
Methylmalonic acid concentration by tissue type over time. (A) MMA concentrations expressed as nmol per gram of tissue. The values are averages from prenatal [embryonic day 19] (N = 3), neonatal [8–12 hour] (N = 3), and metabolic crisis [20–24 hour] (N = 2) mutant animals. Values for age-matched control littermates (N = 2) for each time point are not depicted on this graph but were used to plot the fold change, depicted in additional file 4. The error bars surround the standard deviation for the prenatal time point and the range observed at the stage of metabolic crisis. p-values for the mutant versus age matched controls were <0.01 for all tissues. An asterisk * designates a significant p-value. In the mutants, the MMA content of the liver at 24 hours was significantly greater than skeletal muscle (p = 0.002 at 8 hours, p = 0.05 at 24 hours), brain (p = 0.002 at 8 hours, p = 0.0016 at 24 hours), or kidney (p = 0.0023 at 8 hours, p = 0.0072 at 24 hours). However, only the difference between the liver samples at the 24-hour time point and other tissues at the 8 and 24-hour time points were significant with a p-value less than 0.05.

Organs were examined for MMA content throughout the life of the animals. In the prenatal *Mut *mutant mice, a 19-fold increase in the MMA content of the kidney was observed, with other organs increased only 3–4 fold over wild-type animals (see additional file 4 for a graph of the fold change). At 8 to 12 hours of age, skeletal muscle and kidney MMA levels exceeded the MMA levels of the brain and liver in mutants (Figure 4) but were not significantly greater. At the end of life, however, the MMA content and fold change was increased in the mutant liver, as was the MMA content of the skeletal muscles, kidneys, and brain in descending order (see additional file 4 for a graph of the fold change). Within the affected animal groups, organ specific differences over time were examined for significance using a mixed model ANOVA. First, an effect of time was assessed to examine the hypothesis that MMA concentrations varied over time in an organ specific fashion [F (6,12) = 6.7, p = 0.002]. The effect of time on MMA concentration in each organ type was next determined, with liver [F (2,16) = 43.7, p < 0.0001), skeletal muscle [F(2,16) = 11.8, p = 0.0007) and kidney [F (2,16) = 7.9, p = 0.004) displaying a statistical significant pattern of increasing as time progressed. The changes in brain metabolites [F (2,16) = 2.8, p = 0.09] showed a trend toward increasing over time but did not achieve significance. Next, pair wise contrasts were constructed from the mixed model results in order to compare all tissue types and all sample times. The corresponding p values, adjusted for multiple comparison after bootstrap simulation, were examined for significance using paired t-tests. The most important trend was that the MMA content of the liver at 24 hours was significantly greater than skeletal muscle (p = 0.002 at 8 hours, p = 0.05 at 24 hours), brain (p = 0.002 at 8 hours, p = 0.0016 at 24 hours), or kidney (p = 0.0023 at 8 hours, p = 0.0072 at 24 hours). Furthermore, despite the fact that the skeletal muscle had the highest MMA content at 8 hours, there were no statistically significant differences between organs at 8 hours. Comparison between the mutant versus wild type and heterozygous animals for plasma and tissue metabolites was striking at all points. Because many wild type and heterozygote animals had MMA levels at the lower limit of assay detection, detailed comparisons between these groups were not included in our analysis.

The contribution of other metabolites and cellular processes to the clinical phenotype was also examined. In particular, 2- methylcitrate (2-MC) was elevated in the affected mice in all fluids and tissues examined (data not shown). Both in humans with cobalamin deficiency syndromes [41] and in the mutant mice, the ratio of the diasteromer 2-MCI to 2-MCII was greater in the brain than in the periphery (see additional file 5 for the ratios of the diasteromers in the different tissues over time) as the illness progressed.

### Patient Studies

**Figure 5:**
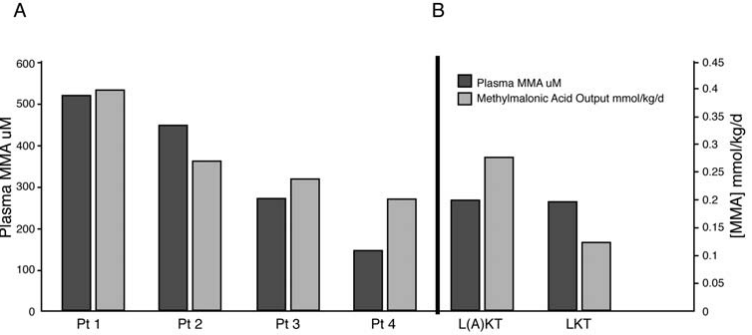
Biochemical parameters in MMA patients. Plasma MMA levels (μM) and 24-hour urine MMA output (mmol/kg/d) in non-transplanted patients (*mut*^*o*^-patients 1–4) with preserved renal function (A) compared to patients who have received solid organ transplants (L(A)KT, LKT) (B). * p-values for the average plasma MMA concentrations and MMA output from the untransplanted patients versus the transplanted patients. Neither the plasma metabolites [F(1,4) = 0.40, p = 0.56] or the MMA output [F (1,4) = 0.70, p = 0.44] was significantly different between the groups.

Whole body output of MMA was estimated from the timed urine collections of six patients with *mut*^*o *^methylmalonic acidemia, including two who had undergone combined liver and kidney transplantation (Figure 5). The two *mut*^*o *^transplant patients, one with an auxiliary liver-kidney transplant (Figure 5, labeled L(A)KT) and one with a replacement liver-kidney transplant (Figure 5, labeled LKT), had nearly identical plasma MMA concentrations, in the 260 μM range (normal < 0.3 μM), when treated with dietary therapy. Compared to other *mut*^*o *^patients with measured glomerular filtration rates greater than 40% predicted for age who were also treated with high calorie, propiogenic precursor restricted diets, the plasma levels of MMA in the transplanted patients approximated that observed in non-transplanted patients, and the 24-hour urinary MMA output [44] (measured in millimoles of MMA in the urine/kg body weight/day) displayed a comparable elevation. The average values for the plasma MMA concentration and MMA output were calculated and compared between the two groups. Neither the plasma metabolites [F(1,4) = 0.40, p = 0.56] or the MMA output [F (1,4) = 0.70, p = 0.44] was significantly different between the groups. The glomerular filtration rate (GFR) was measured with two consecutive 24-hour urine collections and found to be greater than 70 ml/min in the transplanted patients, with normal values of other renal parameters, such as fractional excretion of sodium and urinary amino acids. This indicates that renal dysfunction is not a contributing factor to the methylmalonic acidemia and aciduria observed in these transplanted individuals. Minimal biochemical data was available on the transplant patients when they had preserved renal function and precluded a direct comparison to the effect of combined organ transplantation in each patient.

## Discussion

Our biochemical characterization of methylmalonic acidemia in murine and human systems has provided us with important new insights not examined in studies of a different murine model of methylmalonic acidemia [45]. For example, during gestation (E19) the plasma MMA of *mut*^*o *^mice was in the 170 μM range, much greater than the 30 μM concentration seen in amniotic fluid (urine) samples from mothers known to carry *mut*^*o *^fetuses[46]. These high plasma MMA levels indicate aberrant prenatal metabolism and suggest that the animals may be born partly intoxicated due to the chronicity and magnitude of their exposure to MMA and related metabolites. Prenatal exposure to an abnormal metabolic milieu may explain the onset of clinical symptoms in some of the neonates with *mut*^*o *^methylmalonic acidemia in the first 24 hours of life and the later presentations seen in patients with less severe blocks[4]. *In utero *metabolic changes and the effects on growth and development have become an increasingly recognized feature in disorders of the electron transport chain [47] and future studies of *in utero *metabolism in the *mut*^*o *^mice will examine whether the massive metabolite elevations observed have physiological consequences on energy metabolism.

The pattern of organ MMA concentrations observed as the illness progresses in mice reveals several important trends that can be directly correlated to patients. While the mice are alive and feeding, their plasma MMA concentration is 1.2 mM and the skeletal muscle contains the highest average concentration of MMA per milligram of wet-weight tissue. This suggests that the skeletal muscle is generating significant amounts of MMA. The notion of extra-hepatorenal production of MMA is supported by the studies on the human combined liver-kidney transplant patients, who have massive MMA output (Fig. 5B) and enormously elevated plasma MMA levels comparable to those seen in non-transplanted methylmalonic acidemia patients. Furthermore, because both liver-kidney transplant patients had intact renal function and normal-functioning livers that would metabolize gut-derived propionate, the MMA in these patients must originate principally in other organs.

Direct comparison to the pre-transplant state in solid organ transplant patients was not possible because they had not been studied when renal function was intact nor in the same fashion described in this report. For this reason, the transplant patients were compared to a group of untransplanted patients with the same enzymatic phenotype and preserved renal function who were treated with low protein, high-energy diets. Future efforts to characterize the metabolic parameters in MMA solid organ transplant recipients might benefit from standardized pre- and post- operative regimens to measure metabolic parameters in all patients. Clearly, this will be an important clinical and research problem to address, especially since the number of MMA transplant recipients to date is small but likely to increase as more patients are identified through newborn screening.

In humans, approximately 40 percent of the body mass is skeletal muscle, which plays a major role in branched-chain amino acid catabolism and fatty acid oxidation [48-51]. Because branched-chain amino acid catabolism and odd-chained fatty acid oxidation produce the bulk of the MMA observed in non-transplanted patients[52], the simplest hypothesis to unify the observations from the murine and human studies is that MMA is being generated by the skeletal muscles in both species. Indeed, when methylmalonyl-CoA mutase was previously measured in different murine tissues, skeletal muscle possessed significant holo- and apo- enzyme activity. Furthermore, the promoter was more complex than seen in generic "house-keeping" genes and the Mut gene and protein appeared to be subject to significant regulation at the transcriptional and post-translational levels [53]. To directly examine the skeletal muscle and other tissues for the relative abundance of enzyme, we used Western blot analysis to examine tissue extracts from a wild type mouse and found that the skeletal muscles contained a large amount of immunoreactive enzyme, grossly comparable to the relative amounts present in the kidney and liver (see additional file 6 for a Western analysis of murine tissues). Thus several lines of evidence indicate that the enzyme is expressed, active and abundant in the skeletal muscle.

Skeletal muscle is also an important site of branched-chain amino acid (BCAA) oxidation [50,51], a process suspected to contribute approximately 50 percent of the MMA load observed in humans [52]. The branched-chain alpha-ketoacid dehydrogenase complex is an enzyme involved in branch-chain aminoacid oxidation, which is inhibited via phosphorylation by the branched-chain alpha-ketoacid dehydrogenase kinase [54]. Methylmalonyl-CoA has previously been shown to inhibit branched-chain alpha-ketoacid dehydrogenase kinase activity [55]. In the setting of methylmalonic acidemia, methylmalonyl-CoA accretion may increase the activity of the branched-chain alpha-ketoacid dehydrogenase complex by inhibiting the branched-chain alpha-ketoacid dehydrogenase kinase in the skeletal muscle beds, setting up a physiological forward feeding circuit for MMA production. Increased MMA production through such a mechanism might also contribute to the significant methylmalonic acidemia seen in the transplant patients. The possibility that the MMA concentrations in the tissues of the knock-out mice may be influenced by the developmental regulation for BCAA catabolism will require further examination, especially since the regulation of BCAA catabolism in mice has not been extensively studied. Additionally, the precursors of MMA may vary over time, with odd-chained fatty acids potentially providing a major contribution to the metabolite load in the mice and humans, especially given the evidence from patient studies demonstrating that odd chained fatty acids are increased in patients with propionate metabolic disorders[56,57].

The clinical studies presented here include determination of whole body MMA output in *mut*^*o *^patients, a measurement that has previously been demonstrated to be an effective means to carefully describe whole body MMA metabolism[44]. Previous clinical reports [33,34] and the studies presented here firmly demonstrate that enzymatic correction of the liver and kidney in affected patients by transplantation does not always greatly lower the circulating MMA load. However, liver transplantation patients do realize complete protection from systemic metabolic decompensation. This suggests a tissue-specific response or maladaption to the inherent biochemical lesion in the native liver, and possibly other organs. Our results indicate that modulating metabolism in the skeletal muscle – by gene replacement or pharmacological means – may represent an effective strategy to lower MMA levels and increase metabolic capacity in affected patients. Other disorders of intermediary metabolism that feature impaired branched chain aminoacid oxidation, such as propionic acidemia, might also benefit from these approaches.

## Conclusion

In summary, this report describes the construction and characterization of a new murine model of methylmalonic acidemia and its use to examine organ specific contributions to MMA production in the human condition in the post-transplant state. The targeted allele is null, designed for genomic engineering, and correctable by lentiviral transduction. The combined observations from the murine metabolite studies and patient investigations indicate that during homeostasis, a large portion of circulating MMA has an extra-heptorenal origin and likely derives from the skeletal muscle. Our studies suggest that modulating skeletal muscle metabolism may represent a strategy to increase metabolic capacity in methylmalonic acidemia as well as other organic acidurias. This mouse model will be useful for further investigations exploring disease mechanisms and therapeutic interventions in methylmalonic acidemia, a devastating disorder of intermediary metabolism.

## List of Abbreviations

MMA (methylmalonic acidemia), MUT (human methylmalonyl-CoA mutase), Mut (murine methylmalonyl-CoA mutase), *mut*^*o *^(vitamin B12 non-responsive methylmalonic acidemia), MEF (murine embryonic fibroblasts), CMV (cytomegalovirus), LKT (liver-kidney transplantation), eGFP (enhanced green fluorescent protein), 2-MC (2-methylcitrate), BCAA (branched chain amino acids)

## Competing interests

The author(s) declare that they have no competing interests.

## Authors' contributions

RJC produced reagents, executed experiments, interpreted data and drafted the manuscript; JS coordinated patient care, performed clinical studies and analysis and helped draft the manuscript; HF produced recombinant ES cells; MT performed experiments; SS performed metabolite analysis and interpretation; RA performed metabolite analysis and interpretation; KHK produced recombinant ES cells and helped draft the manuscript; HHK aided with data analysis and helped draft the manuscript; CPV conceived the knock-out model, produced reagents, executed experiments, performed clinical studies, interpreted data and drafted the manuscript. All authors read an approved the final version of the manuscript.

## Pre-publication history

The pre-publication history for this paper can be accessed here:



## Supplementary Material

Additional file 1 Genotype and screening assays. (A). A 2% agarose gel showing the results of the genotyping reaction across the 5' loxP site. Lanes 1–4, 6, and positive control are from heterozygote animals and exhibit two bands: 190 bp (wild-type) and 225 bp, which contains the flank sequences as well as the loxP site. Lane 5, homozygous for the wild-type *Mut *locus, has a single wild-type band while lane 7, a homozygous *Mut *knock-out, has a single loxP site. Para-nitroanaline (PNA) reactivity of 3.5 μl of urine from each animal yields an emerald green positive reaction for the *Mut *mutant. The bottom of the panel shows the standards for the PNA reaction: A-10 mM, B-100 mM, C-1 mM, D-blank. The affected animal (number 7) appears to have a urinary MMA concentration between 10 and 100 mM. Click here for file

Additional file 2 Primers for PCR and RT-PCR Click here for file

Additional file 3 Western analysis of wild-type and Mut embryonic fibroblast extracts. Western blotting using anti-mutase antisera reveals a band of ~80 kd in the wild-type extracts that is completely absent from the *Mut *null MEF line. Recombinant murine methylmalonyl-CoA mutase expressed in yeast served as a positive control (Y) and is located next to the marker (M) lane. The sizes of the molecular weight standards in kilodaltons are indicated. Anti-actin antibodies were used to control for the amount of protein loaded per well. Click here for file

Additional file 4 Methylmalonic acid fold change by tissue type. The values are averages from prenatal [embryonic day 19] (n = 3), neonatal [8–12 hour] (n = 3) and metabolic crisis [20–24 hour] (n = 2) affected animals. The fold change is plotted for each time point compared to control littermates (n = 2). Click here for file

Additional file 5 2-Methylcitrate I/II Ratios in Mouse Organs Click here for file

Additional file 6 Tissue distribution of murine methylmalonyl-CoA mutase. Western analysis of tissue extracts prepared from a wild-type mouse. 10 μg of total protein were loaded in each lane and probed with anti-mutase antibodies or anti-actin antibodies. A recombinant mouse methylmalonylCoA mutase protein (labeled yeast) served as the positive control. The marker lane (M) and the sizes of the molecular weight standards in kilodaltons are indicated. Click here for file
