# Supplementary figures and images for: Metabolic phenotype of methylmalonic acidemia in mice and humans: the role of skeletal muscle
Source: BMC Med Genet. 2007 Oct 15;8:64. doi: 10.1186/1471-2350-8-64 (PMC2140053; doi:10.1186/1471-2350-8-64)

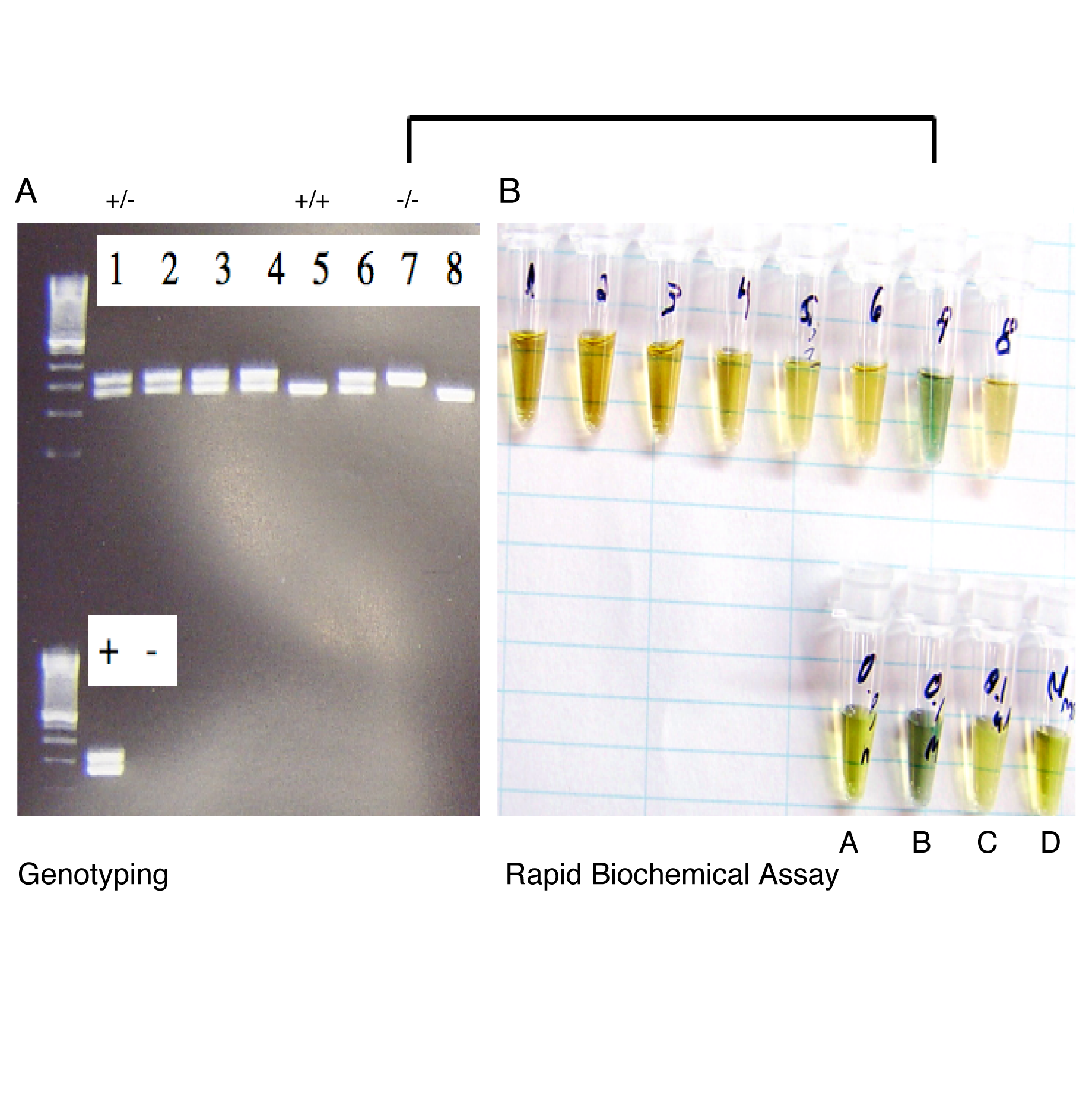

Supplement: Additional file 1 — Genotype and screening assays. (A). A 2% agarose gel showing the results of the genotyping reaction across the 5' loxP site. Lanes 1–4, 6, and positive control are from heterozygote animals and exhibit two bands: 190 bp (wild-type) and 225 bp, which contains the flank sequences as well as the loxP site. Lane 5, homozygous for the wild-type Mut locus, has a single wild-type band while lane 7, a homozygous Mut knock-out, has a single loxP site. Para-nitroanaline (PNA) reactivity of 3.5 μl of urine from each animal yields an emerald green positive reaction for the Mut mutant. The bottom of the panel shows the standards for the PNA reaction: A-10 mM, B-100 mM, C-1 mM, D-blank. The affected animal (number 7) appears to have a urinary MMA concentration between 10 and 100 mM. [file 1471-2350-8-64-S1.tiff]

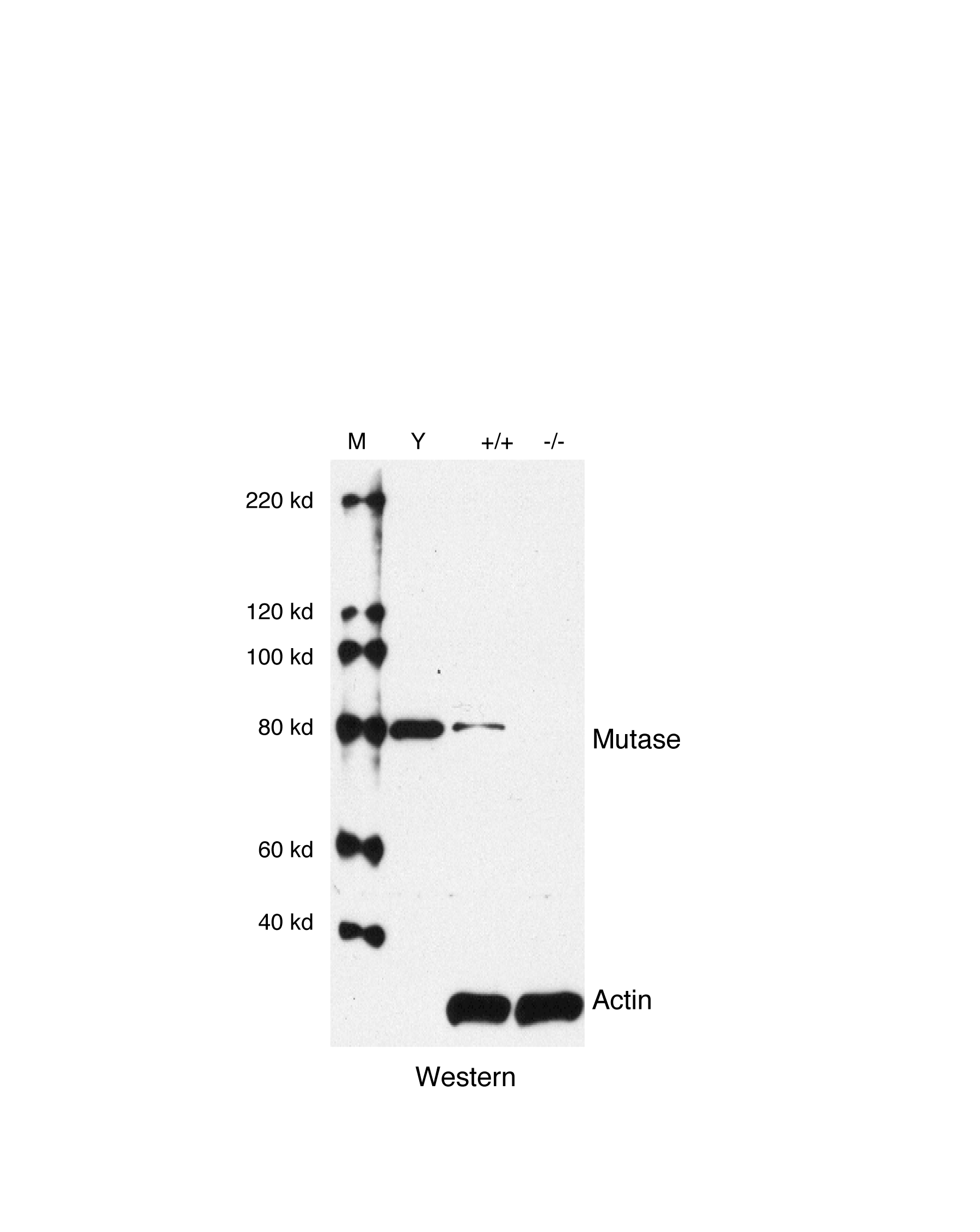

Supplement: Additional file 3 — Western analysis of wild-type and Mut embryonic fibroblast extracts. Western blotting using anti-mutase antisera reveals a band of ~80 kd in the wild-type extracts that is completely absent from the Mut null MEF line. Recombinant murine methylmalonyl-CoA mutase expressed in yeast served as a positive control (Y) and is located next to the marker (M) lane. The sizes of the molecular weight standards in kilodaltons are indicated. Anti-actin antibodies were used to control for the amount of protein loaded per well. [file 1471-2350-8-64-S3.tiff]

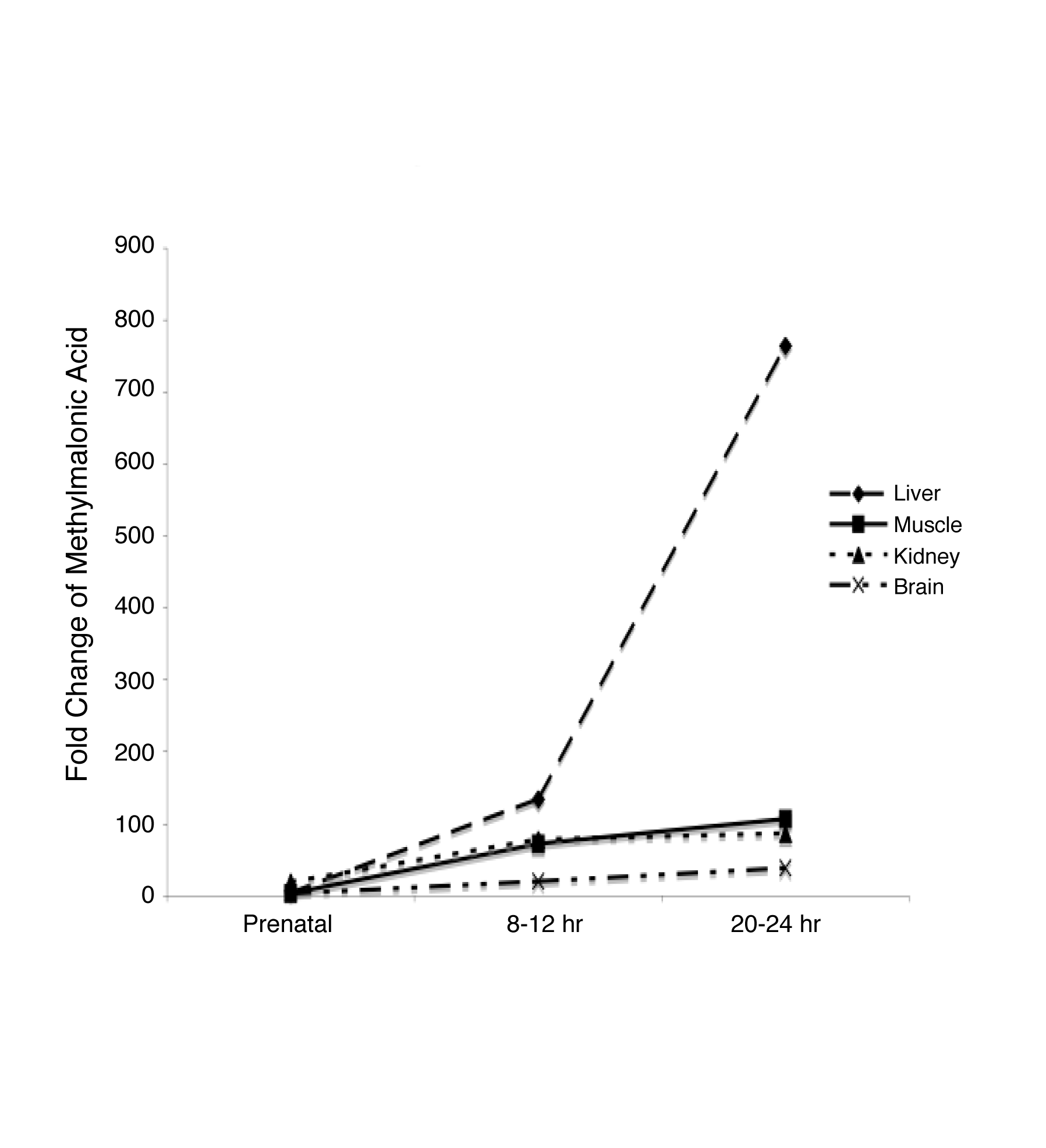

Supplement: Additional file 4 — Methylmalonic acid fold change by tissue type. The values are averages from prenatal [embryonic day 19] (n = 3), neonatal [8–12 hour] (n = 3) and metabolic crisis [20–24 hour] (n = 2) affected animals. The fold change is plotted for each time point compared to control littermates (n = 2). [file 1471-2350-8-64-S4.tiff]

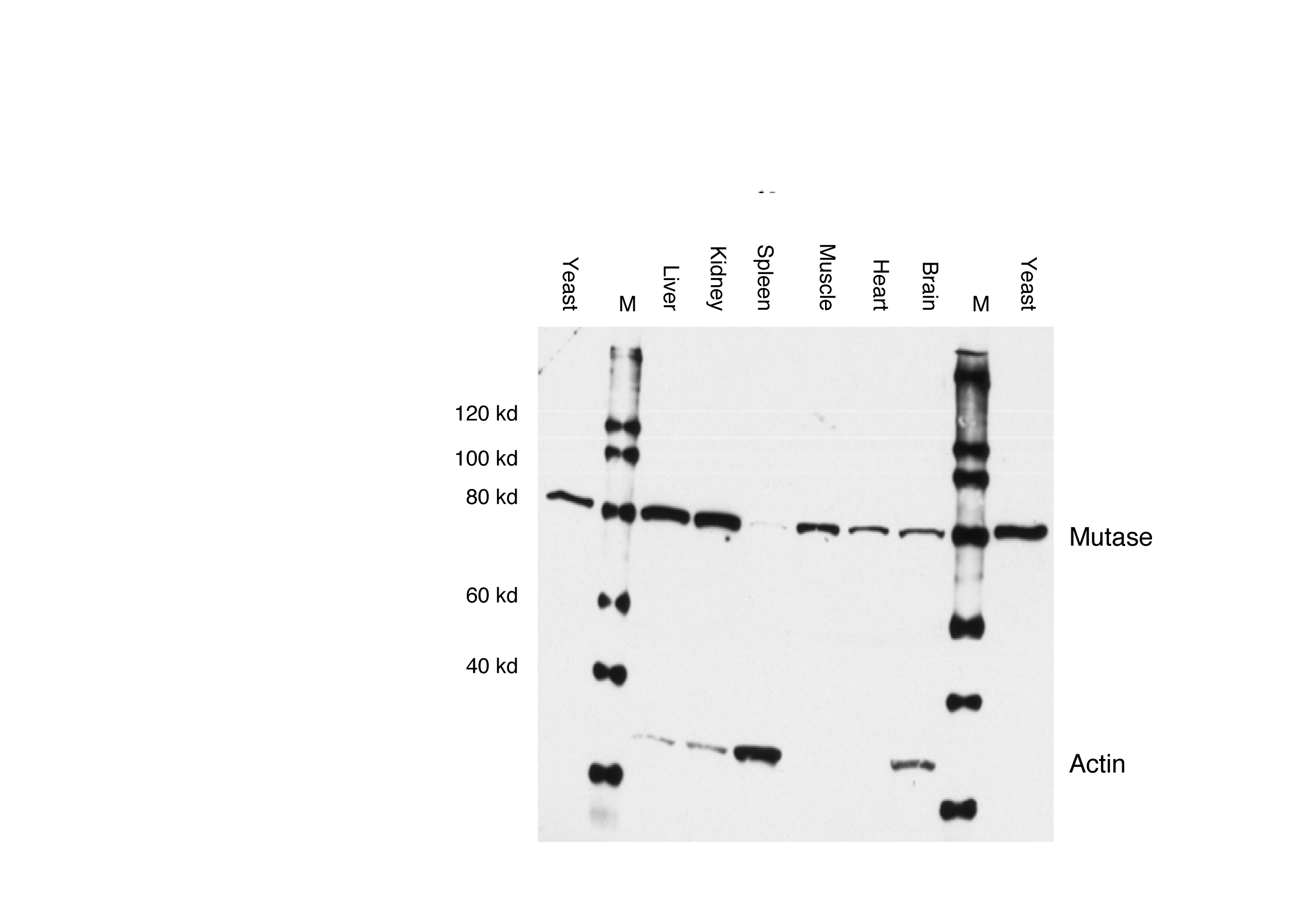

Supplement: Additional file 6 — Tissue distribution of murine methylmalonyl-CoA mutase. Western analysis of tissue extracts prepared from a wild-type mouse. 10 μg of total protein were loaded in each lane and probed with anti-mutase antibodies or anti-actin antibodies. A recombinant mouse methylmalonylCoA mutase protein (labeled yeast) served as the positive control. The marker lane (M) and the sizes of the molecular weight standards in kilodaltons are indicated. [file 1471-2350-8-64-S6.tiff]
