# Supplementary material for: Metabolic phenotype of methylmalonic acidemia in mice and humans: the role of skeletal muscle
Source: BMC Med Genet. 2007 Oct 15;8:64. doi: 10.1186/1471-2350-8-64 (PMC2140053; doi:10.1186/1471-2350-8-64)
Supplement: Additional file 2 — Primers for PCR and RT-PCR [file 1471-2350-8-64-S2.pdf]

Primers for PCR and RT-PCR

Primer Name	Sequence (5'-3')
Lox P 5' (genotyping primer A)	ccattctgggaaggcttcta
Lox P 3' (genotyping primer B)	tgcacagagtgctagtttcca
Exon 2 Mut 5' (RT-PCR primer)	gaccgttctcatttccttttgg
Exon 6 Mut 3' (RT-PCR primer)	cttcagagcagcctcataaacgtc
Exon 3 Mut (RT-PCR primer)	cagttctaaaatggcatcagtcc
Exon 4 Mut (RT-PCR primer)	gcaggctttagtactgtggaaga
GAPDH 5' (RT-PCR primer)	tgaaggtcggtgtgaacggatttgg
GAPDH 3' (RT-PCR primer)	catgtaggccatgaggtccaccac
