# Supplementary material for: Metabolic phenotype of methylmalonic acidemia in mice and humans: the role of skeletal muscle
Source: BMC Med Genet. 2007 Oct 15;8:64. doi: 10.1186/1471-2350-8-64 (PMC2140053; doi:10.1186/1471-2350-8-64)
Supplement: Additional file 5 — 2-Methylcitrate I/II Ratios in Mouse Organs [file 1471-2350-8-64-S5.pdf]

2-Methylcitrate I/II Ratios in Mouse Organs

	Brain	All other tissue		P-value
Wild type	1.32	1.67	n=2	NS
Prenatal Muto	1.26	1.38	n=3	NS
Neonatal Muto	2.01	1.57	n=3	0.001
Metabolic decompensation, Muto	2.04	1.41	n=2	0.0002
